# Takotsubo Cardiomyopathy in Epilepsy: A Systematic insight into the Existing Literature from 2000-2023

**DOI:** 10.12669/pjms.40.12(PINS).11274

**Published:** 2024-12

**Authors:** Haseeb Mehmood Qadri, Fiza Ismail, Momina Khawaja, Amna Sohail, Seeneen Bukhari, Asif Bashir

**Affiliations:** 1 Dr. Haseeb Mehmood Qadri, Punjab Institute of Neurosciences, Lahore, Punjab, Pakistan; 2 Dr. Fiza Ismail Continental Medical College, Lahore, Punjab, Pakistan.; 3 Dr. Momina Khawja Gulf Medical University, United Arab Emirates.; 4 Dr. Amna Sohail Chengde Medical University, China.; 5 Seeneen Bukhari Ameer-ud-Din Medical College, Lahore, Punjab, Pakistan.; 6 Professor Asif Bashir, Punjab Institute of Neurosciences, Lahore, Punjab, Pakistan

**Keywords:** Stress Cardiomyopathy, Sudden unexpected death in epilepsy, Left Ventricular Apical Ballooning Syndrome

## Abstract

**Background & Objective:**

Takotsubo cardiomyopathy (TCM), manifests as left ventricular dysfunction triggered by physical or emotional stress. It leads to higher morbidity in epileptic patients and can progress to complications. To find out the correlation between Takotsubo cardiomyopathy and epilepsy and to investigate pathophysiology and associated types of epilepsy.

**Methodology:**

This systematic review adhered to PRISMA guidelines and was sourced from the PubMed Central database. Search terms were pertinent to cardiomyopathy and epilepsy. Sixteen studies, comprising case reports and a case series were selected from 2000 to 2023 for data extraction. The quality evaluation was executed via the Joanna Briggs Institute Critical Appraisal checklist.

**Results:**

The review included 18 female patients with a mean age of 57.22 years. Predominant symptoms included tonic-clonic seizures (66.66%). Seizure-induced TCM pathophysiology implicates catecholamine surge, precipitating myocardial stunting and characteristic apical ballooning. Most patients had a history of epilepsy (38.88%). ECG findings showed tachycardia (38.88%) and ST-segment elevation (38.88%). Elevated troponin levels were noted in 83.33% of patients. Echocardiography showed reduced ejection fraction (72.22%), hypokinesia (38.88%) and akinesis (27.77%). Treatment involved Benzodiazepines (50%), Beta-blockers (61.11%) and Phenytoin (38.88%). The majority of patients showed improvement in echocardiography findings (55.55%) and ECG findings (11.11%).

**Conclusion:**

TCM in epilepsy patients evinces significant female predominance, with pathophysiology rooted in seizure-induced catecholamine surge. Early recognition in high-risk patients is essential in preventing complications.

## INTRODUCTION

Takotsubo cardiomyopathy (TCM) is characterized by a temporary and reversible dysfunction of the left ventricle apex that resembles a myocardial infarction (MI) in the absence of coronary artery disease. The hallmark of this syndrome is the apical ballooning of the left ventricle, observed during systole, which resembles a Takotsubo—a Japanese octopus trap—giving the condition its name.^1^ This condition predominantly affects postmenopausal women and is often precipitated by episodes of intense emotional or physical stress.^2^ TCM was initially considered self-resolving however, it is now considered to be associated with substantial short and long-term morbidity and mortality.^3^ The precise pathophysiological mechanisms remain a subject of ongoing research, nevertheless, there have been instances where epileptic seizures have precipitated TCM. Moreover, TCM may precipitate life-threatening complications in post-ictal phase, emphasizing the importance of understanding the pathophysiology of TCM in epilepsy.^4^ The main aim of this review was to explore the connection between TCM and epilepsy, particularly investigating the pathophysiological processes of TCM induced by epileptic seizures.

To the best of our knowledge, this is the first systematic review that explores the intricate relationship between TCM and epilepsy that aimed to elucidate the underlying pathophysiological mechanisms, identify patient risk factors, and provide insights into diagnosis and clinical management strategies. This comprehensive synthesis of existing research could also highlight knowledge gaps, guiding future studies and potentially influencing healthcare strategies for better monitoring and management of patients with epilepsy to prevent TCM occurrences and enhance patient care.

## METHODS

A systematic review on the topic of ‘Epilepsy in Takotsubo Cardiomyopathy’ is conducted as per guidelines of Preferred Reporting Items for Systematic Reviews and Meta-Analysis (PRISMA). This systematic review was registered with PROSPERO (CRD42024507100). PubMed Central was used to gather case series and case reports for inclusion. Quality assessment of articles was performed. The Boolean search strategy employed terms such as “Cardiomyopathy” AND “Epilepsy”, “Cardiomyopathy” AND “Seizure”, “Cardiomyopathy” AND “Convulsion” and “Cardiomyopathy” AND “Fits”.

### Eligibility Criteria:

Inclusion criteria comprised case reports and case series, studies on living humans and studies that were in the English language. Exclusion criteria included Letters to the editors, editorials, systematic review and meta-analysis, animal studies, cadaveric studies and non-English studies.

### Data Extraction:

Independent reviewers extracted data in accordance with the following variables: Title, abstract, methods, and main results. Data accuracy and completeness were analyzed and verified. Final results were compiled using Microsoft Office 365 Word-Generated proforma.

### Study Selection:

**Fig.1:**
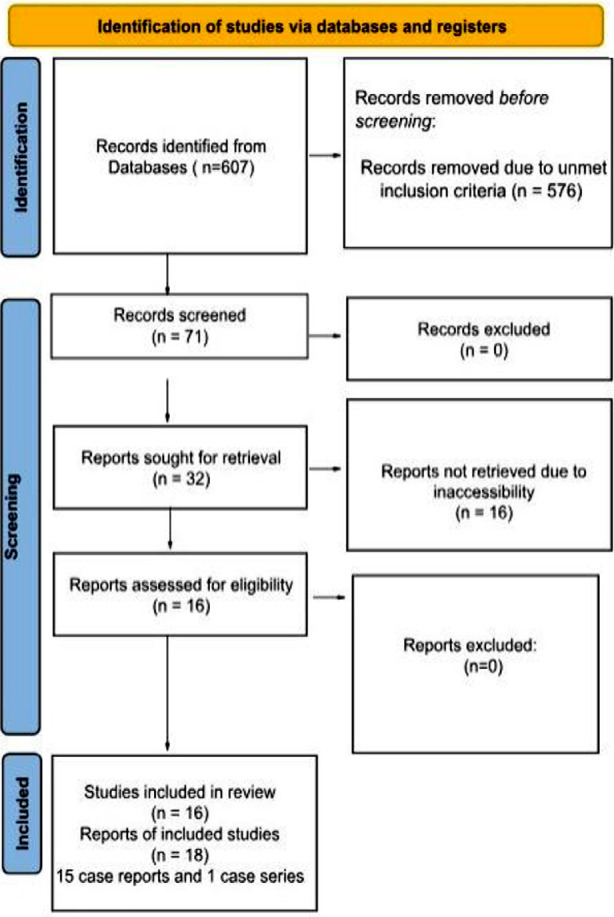
Preferred Reporting Items for Systematic Review and Meta-Analyses (PRISMA) flow chart for search strategy and the quality assessment for included studies.

Fifteen case reports and one case series comprising a total of sixteen out of twenty-six articles from 2000 to 2023 were included. The PRISMA flowchart is illustrated below (Fig.1).

### Quality Assessment:

The quality of the included studies was evaluated using Joanna Briggs Institute (JBI) Critical Appraisal Checklist for case-control studies. The JBI Checklist is mentioned in Appendices.

A summary of the literature included in this systematic review is given in Table-I:

**Table-I:** Summary of findings in patients of Takotsubo Cardiomyopathy.

Study by	Parameters				

	Title	Gender/ Age	Past medical history	ECG Findings	Type of seizure
Harb et al. 4	Takotsubo Syndrome (Broken-Heart Syndrome or Stress Cardiomyopathy) in an Epileptic Pregnant Woman: A Case Report	F/33 y	Focal epilepsy	Sinus tachycardia, Global non-specific ST-T changes	Generalized tonic-clonic seizure
Yazdi et al.^5^	Seizure-Induced Cardiomyopathy: A Case of Takotsubo Cardiomyopathy Following an Epileptic Event	F/75 y	Hypertension, Epilepsy, Coronary artery disease (CAD)	Supraventricular tachycardia	Tonic-clonic seizure
Saouma et al.^6^	Takotsubo cardiomyopathy following a simple partial seizure	F/67 y	Familial meningiomatosis,	ST elevation (DI and aVL)	simple partial seizure
Conte et al.^7^	Seizure-Associated Takotsubo Cardiomyopathy	F/48 y	Encephalomalacia, Arteriovenous malformation, Coagulopathy	Inferior non - non-ST-segment elevations	Generalized tonic-clonic seizure
Nandal et al.^8^	Takotsubo cardiomyopathy triggered by status epilepticus: case report and literature review	F/71 y	Right temporal parietal craniotomy, Intracranial hemorrhage (ICH) , Epilepsy	Lateral ST elevation (I, aVL)	Generalized tonic-clonic seizure
Prosperi-Porta et al.^9^	Diffuse Deep T-Wave Inversions Following a Generalized Seizure	F/44 y	Infectious endocarditis	Sinus bradycardia, persistent left anterior fascicular block, deep T-wave inversions in limb leads I, II, aVF, aVL, and aVR, and precordial leads V1-V6	Generalized tonic-clonic seizure
Miller et al.^10^	Recurrence of Postoperative Stress-Induced Cardiomyopathy Resulting from Status Epilepticus	F/49 y	Hypertension, Refractory status epilepticus, Endometrioid endometrial carcinoma	Normal sinus rhythm (NSR) and right bundle branch block (RBBB)	Generalized tonic-clonic seizure
Simsek et al.^11^	Unusual combined cause of Takotsubo cardiomyopathy: Hyponatremia and seizure	F/69 y	Hypertension, Epilepsy	Sinus rhythm with diffuse upsloping 1 mm ST-segment elevation	Generalized tonic–clonic seizure
Koo et al.^12^	Biventricular Takotsubo Cardiomyopathy Associated with Epilepsy	F/83 y	Epilepsy after cerebral hemorrhage	Precordial V2-4 ST segment elevation	Generalized tonic–clonic seizure
Kyi et al.^13^	Seizure Associated Takotsubo Syndrome: A Rare Combination	F/61 y	Epilepsy, Bipolar disorder, Hypertension	ST elevation in anterolateral leads	Generalized Tonic-clonic seizure
Naganuma et al.^14^	Epilepsy and Takotsubo Cardiomyopathy: A Case Report	F/60 y	Epilepsy, Mitral valve replacement, Subarachnoid hemorrhage (SAH), Aneurysm clipping	Negative T waves in II, III, aVF, and V2-6	Generalized Tonic seizure
Dupuis et al.^15^	Takotsubo syndrome (TKS): A possible mechanism of sudden unexplained death in epilepsy (SUDEP)	F/50 y	-	Antero-septal Q waves	Tonic-clonic seizure after a partial seizure
Rocha et al.^16^	Takotsubo cardiomyopathy: a rare, but serious, complication of epileptic seizures	F/44 y	Subarachnoid hemorrhage, Coil embolization	Sinus tachycardia, T wave inversion in V1–V3 leads	Generalized Tonic-clonic seizure
Legriel et al. 17	Recurrent Takotsubo Cardiomyopathy Triggered by Convulsive Status Epilepticus	F/54 y	Depression, Epilepsy, Stroke	Sinus tachycardia with T-wave inversion	Partial CSE (convulsive status epilepticus)
Sakuragi et al.^18^	A case of takotsubo cardiomyopathy associated with epileptic seizure: reversible left ventricular wall motion abnormality and ST-segment elevation	F/59 y	Pacemaker Implantation for sick sinus syndrome, Astrocytoma	ST-segment elevation in leads of I, aVL, and V2–4, QTc interval was 467ms, deep inverted T waves with prolongation of the QTc to 515ms	-
Lemke et al.^19^	Tako-Tsubo Cardiomyopathy Associated with Seizures	F/63 y F/5o y	Case 1: schizophrenia,	Case 1: sinus tachycardia with ST elevations in V2 and V3 Case 2: Sinus tachycardia, ST depression in V1–3 leads Case 3: sinus tachycardia with ST depression.	Case 1: Tonic-clonic seizure Case 2: Generalized tonic-clonic seizure Case 3: Generalized tonic-clonic seizure

## RESULTS

This study includes fifteen case reports and one case series, with the majority of cases from the USA (n=5) and Japan (n=2) (Table-II). Additional cases were reported from UAE, Lebanon, Australia, Portugal, France, Canada, Korea, Turkey, and Belgium. Most patients were from the USA (n=7) and Japan (n=2). All patients were female with a mean age of 57.22 ± 12.86 years.

**Table-II:** Total number of patients suffering from TCM.

Study type	Number of studies	Number of patients
Case report	15	15
Case series	1	3
Total	16	18

Majority of the patients presented with seizures (77.77%), encompassing generalized epileptic seizure (33.33%), status epilepticus (22.22%), unclassified tonic-clonic seizures (16.66%) and simple partial seizure (5.55%). Other patients presented in a post-ictal state (5.55%) and altered state of consciousness (Table-III).

**Table-III:** Presenting symptoms in patients suffering from TCM.

Presenting symptom	Number of cases, n (N=18)	Percentage occurrence n/N, %
Generalized epileptic seizure	6	33.33%
Status epilepticus	4	22.22%
Unspecified tonic-clonic seizure	3	16.66%
Admitted for management of other disease (sepsis, hysterectomy)	2	11.11%
Simple partial seizure	1	5.55%
Altered consciousness	1	5.55%

The predominant signs depicting the cardiac events were hypotension (22.22%) followed by respiratory distress (16.66%) including tachypnea, decreased spo2 as well as tachycardia, additional heart sounds and chest pain. Other postictal signs observed in patients were neurological deficit (16.66%), and conscious disturbances (11.11%) including low GCS, Todd’s paresis and gaze deviation (Table-IV).

**Table-IV:** Signs in patients suffering from TCM.

	Presenting sign	Number of cases, n (N=18)	Percentage occurrence n/N, %
Signs associated with TCM	Hypotension	4	22.22%
	Respiratory distress	3	16.66%
	Tachycardia	2	11.11%
	S3 sound/ gallop rhythm	2	11.11%
	Chest pain	2	11.11%
Signs associated with seizure	Post-ictal neurological deficit (Hemiparesis, gaze deviation)	3	16.66%
	Post-ictal confusion and altered consciousness	2	11.11%

Past medical histories contributing to the development of seizures include epilepsy in 38.88% of patients, cerebrovascular accident including ICH (22.22%) and stroke (5.55%), hypertension (22.22%) and a prior history of neurosurgical intervention (16.66%) (Table-V). ECG findings indicated Tachycardia (38.88%), ST-segment elevation (38.88%), T-wave inversion (27.77%) in five patients and other variations (Table-VI).

**Table-V:** Past medical history of patients suffering from TCM.

Past medical history	No. of cases, n (N=18)	Percentage occurrence n/N, %
Epilepsy	7	38.88%
Hypertension	4	22.22%
Intracranial hemorrhage (ICH)	4	22.22%
Neurosurgery	3	16.66%
Psychiatric conditions (Depression, schizophrenia, bipolar disorder)	3	16.66%
Encephalomalacia	1	5.55%
Arteriovenous malformation	1	5.55%
Coagulopathy	1	5.55%
Astrocytoma	1	5.55%
Stroke	1	5.55%
Aneurysm	1	5.55%
Deep vein thrombosis	1	5.55%

**Table-VI:** ECG finding in patients presented with TCM.

ECG finding	Number of cases, n (N=18)	Percentage occurrence n/N, %
Tachycardia	7	38.88%
ST-segment elevation	7	38.88%
T-wave inversion	5	27.77%
ST-segment depression	2	11.11%
Normal sinus rhythm	2	11.11%
Non-st segment elevation	1	5.55%
Bradycardia	1	5.55%
Antero-septal Q waves	1	5.55%
QTc prolongation	1	5.55%
Right bundle branch block	1	5.55%

EEG was done after the postictal period during hospital admission. It displays diverse abnormalities in a subset of patients, the majority of EEG findings showed no epileptiform discharges while 5.55% population had bilateral slow waves, irritative discharges, severe diffuse background slowing, mild generalized slowing, pharmacological beta activity, and excessive beta activity in each patient (Table-VII). Transthoracic echocardiography was done in 88.88% of the population, while chest X-ray was done in 27.77% of the population. 16.66% population had CT chest and 5.55% population underwent brain CT and 1231-MIBG (Table-VIII).

**Table-VII:** EEG finding in patients of TCM.

EEG findings	Number of cases, n	Percentage occurrence n/N, %
No epileptiform discharges	5	27.77%
Bilateral slow waves with irritative discharges	1	5.55%
Slow sharp wave activity	1	5.55%
Severe diffuse background slowing	1	5.55%
Mild generalized slowing	1	5.55%
Pharmacological beta activity	1	5.55%
Excessive beta activity	1	5.55%

**Table-VIII:** Radiological investigation in patients presented with TCM.

Radiological investigation	Number of cases, n	Percentage occurrence n/N, %
Transthoracic echocardiography	16	88.88%
CXR	5	27.77%
CT Chest	3	16.66%
Brain CT	1	5.55%
1231-MIBG	1	5.55%

CXR = chest x ray, CT = computed tomography, MIBG = iodine meta-iodobenzylguanidine.

Most common Echocardiography findings included reduced ejection fraction ranging 15-51% (72.22%), wall abnormalities (hypokinetic apical segment 38.88%, akinesis 27.77% and dyskinesias 22.22%, and hyperkinestic basal segment 11.11% (Table-IX). Elevated troponin levels were noted in 83.33% of patients with an average troponin level of 7.14 ng/ml, along with raised BNP levels and leukocyte count (11.11%). 11.11% of patients had metabolic acidosis while CKMB and lactate dehydrogenase were raised in the 5.55% population (Table-X).

**Table-IX:** Echocardiography findings in patients presented with TCM.

Echocardiography findings	No. of cases n, (N=18)	Percentage occurrence n/N, %
Reduced ejection fraction	13	72.22%
Hypokinetic apical segment	7	38.88%
Apical inferior or inferolateral Akinesia	5	27.77%
Apical ballooning/dyskinesis	4	22.22%
Hyperkinetic basal segment	2	11.11%
Mitral regurgitation	1	5.55%
Tricuspid regurgitation	1	5.55%

**Table-X:** Hematological findings in patients presented with TCM.

Hematological investigation done	No. of cases, n	Percentage occurrence n/N, %
Raised troponin level	15	83.33%
Raised pro BNP	2	11.11%
Elevated leukocyte count	2	11.11%
Metabolic Acidosis	2	11.11%
CK-MB	1	5.55%
Lactate dehydrogenase	1	5.55%

The predominant seizure type was tonic clonic (72.22%) in the studied patients (Table-XI). The majority of patients (44.44%) reported no complications. However, 16.66% of patients developed cardiogenic shock and pulmonary edema (Table-XII).

**Table-XI:** Types of seizure in patients presented with TCM.

Type of seizure	No. of cases, n	Percentage occurrence n/N, %
Generalized tonic-clonic	13	72.22%
Simple partial	2	11.11%
Partial CSE	1	5.55%

CSE= conv.

**Table-XII:** Complications in patients presented with TCM.

Complications	No. of cases, n	Percentage occurrence n/N, %
No complication	8	44.44%
Cardiogenic shock	3	16.66%
Pulmonary edema	3	16.66%
Hypertension	1	5.55%

Treatment modalities involved the management of neuropathic and Cardiac symptoms. Neuropathic symptoms were treated with anti-convulsant drugs such as benzodiazepines in 50% of patients (Diazepam was given to 22.22% population, lorazepam to 11.11%, midazolam and clobazam to 5.55% population). Other given drugs for neurological symptoms were phenytoin in 38.88% population and sodium valproate in 33.33% population. Levetiracetam to 27.77%, carbamazepine to 16.66% and magnesium sulfate to 5.55% (Table-XIII).

**Table-XIII:** Anticonvulsant drugs used in the treatment.

Treatment in response to neuropathic symptoms	No. of cases, n	Percentage occurrence n/N, %
Benzodiazepines	9	50%
Phenytoin	7	38.88%
Sodium valproate	6	33.33%
Levetiracetam	5	27.77%
Carbamazepine	3	16.66%
Magnesium sulfate	1	5.55%

Beta-blockers were the primary treatment for cardiac issues (61.11%), with propranolol and metoprolol being the most commonly used beta-blockers (11.11%). ACE inhibitors were given to 33.33% of patients. Anti-epileptic drugs, aspirin, and inotropes were also used (Table-XIV). Following treatment 55.55% showed improvement in their echocardiographic findings and 11.11% showed ECG findings and no outcome was specified in 27.77% of patients (Table-XV).

**Table-XIV:** Hospital management in patients presented with TCM.

Treatment in response to cardiac symptoms	No. of cases n, (N=18)	Percentage occurrence n/N, %
B blocker	11	61.11%
ACE inhibitors	6	33.33%
Inotropes	4	22.22%
Aspirin	4	22.22%
Heparin drip	2	11.11%
Thiamine/folate	1	5.55%

**Table-XV:** Improvement in Radiological and hematological findings after treatment in patients presenting with TCM

Improvement in radiological changes	No. of cases n (N=18)	Percentage occurrence n/N, %
Improvement in Echographic findings (LVEF)	10	55.55%
Improvement in ECG findings	2	11.11%
Treatment not specified	5	27.77%

## DISCUSSION

Takotsubo cardiomyopathy (TCM) is characterized by transient and reversible systolic abnormality affecting the apex of the left ventricle, resembling myocardial infarction (MI) despite the absence of coronary artery disease (CAD).^1^ TCM is known to be triggered by physical or emotional stress 7 and also correlated with epilepsy.^5^ TCM may lead to reduced myocardial contractility, resulting in cardiogenic shock, cardiac arrhythmia, and SUDEP.^20^ Due to higher incidence of perinatal risk factors, and a higher rate of CNS infection epilepsy in more common lower middle-income countries which predispose patients to complications like TCM.^21^

### Pathophysiology:

**Fig.2:**
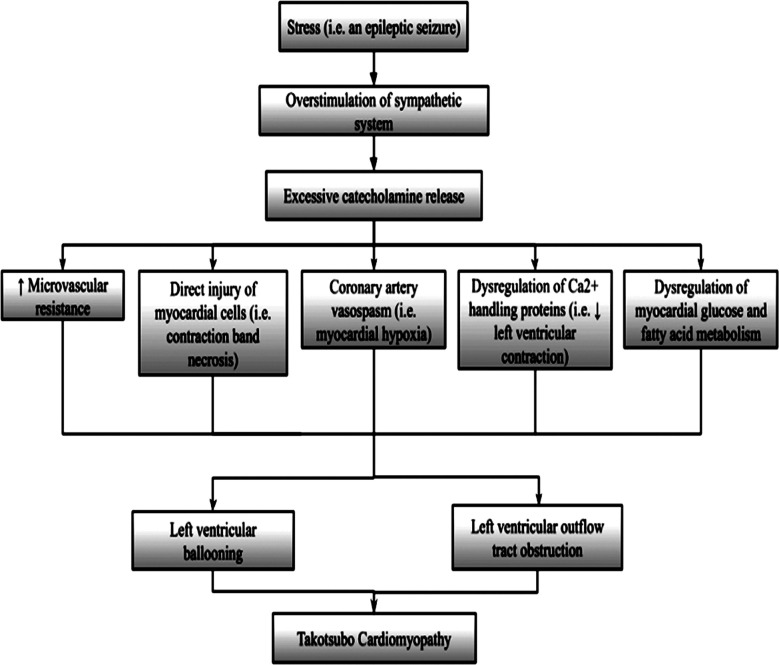
Pathogenesis of Takotsubo cardiomyopathy.

Though its underlying pathophysiology remains unknown, several etiologies have been suggested, with the most commonly proposed explanation for seizure-associated Takotsubo Cardiomyopathy being the excessive release of catecholamines following an epileptic seizure, which causes transient stress-induced demand ischemia.^21^ Following a seizure, plasma levels of epinephrine and norepinephrine surge within 30 minutes, subsequently decreasing rapidly. The norepinephrine surge, from widespread sympathetic neural activation leads to direct vasoconstriction. Meanwhile, the epinephrine spike leads to cardiovascular implications.^22^ Epinephrine storm predominantly targets the beta two receptors on the apical segments of ventricles thus producing the characteristic dilatation of the left ventricle with subsequent left ventricular dysfunction.^6^ Moreover, high epinephrine levels directly injure the myocardial cells via calcium leakage due to hyperphosphorylation of the ryanodine receptor exacerbating cardiotoxicity with infiltration of inflammatory cells, fibrosis and contraction band necrosis.^1^ Other factors like coronary artery vasospasm and microcirculatory failure are also involved.^13^ In addition to that, Positron Emission Tomography (PET) studies have likely suggested defects in myocardial glucose and fatty acid metabolism causing a derangement affecting ventricular wall motility.^23^ One theory proposes that autonomic dysfunction is due to focal epileptic discharge in the temporal lobe resulting in cardiac injury (Fig.2).^16^

**Fig.3:**
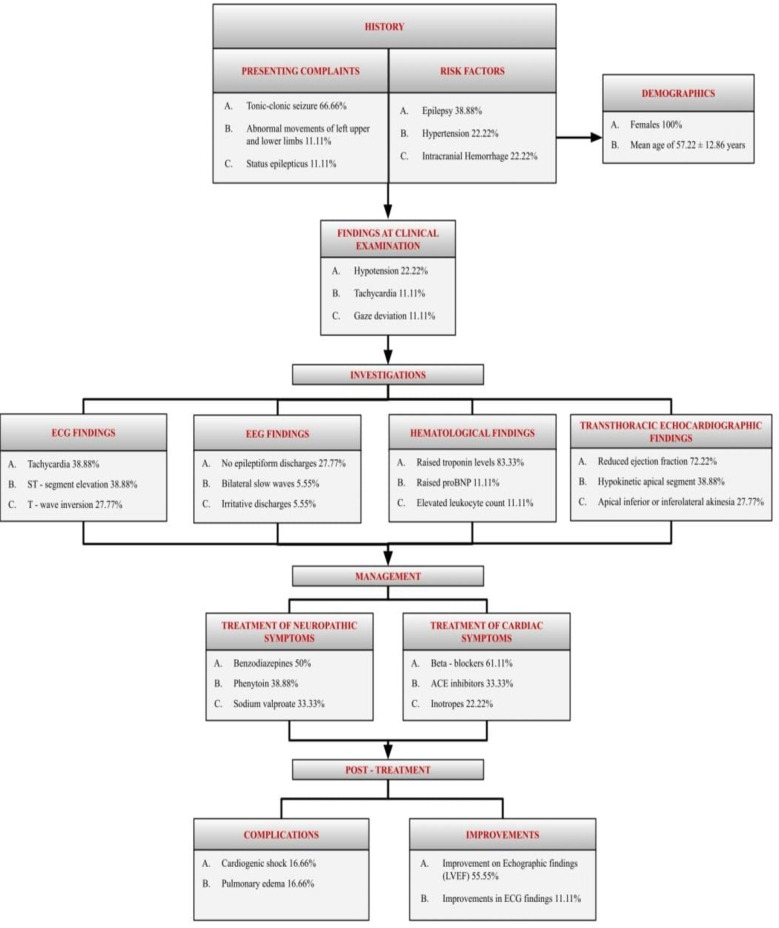
A Summary of findings in Takutsubo cardiomyoathy induced by epilepsy.

### Gender predisposition:

The study of the aforementioned cases also brought attention to the higher occurrence of TCM in women than in men, especially in post-menopausal women. Similar to the study by Contreras Gutiérrez et al. suggesting the majority of patients (82—100%) were women with an average age of 62—75 years.^23^ One mechanism for this increased incidence could be decreased estrogen, which is believed to desensitize the heart’s reaction to catecholamines, and there may be rebound cardiac hypersensitivity following menopause.^19^

## APPENDICES

**Table-XVI:** JBI critical Appraisal checklist for the included case reports, where Y: Yes and N: No.

Author	Q1	Q2	Q3	Q4	Q5	Q6	Q7	Q8	Overall Appraisal
Harb et al.^4^	Y	Y	Y	Y	Y	Y	Y	Y	Included
Yazdi et al.^5^	Y	Y	Y	Y	Y	Y	Y	Y	Included
Saouma et al.^6^	Y	Y	Y	Y	Y	Y	N	Y	Included
Conte et al.^7^	Y	Y	Y	Y	Y	Y	N	Y	Included
Nandal et al.^8^	Y	Y	Y	Y	Y	N	N	Y	Included
Prosperi-Porta et al.^9^	Y	Y	Y	Y	Y	Y	N	Y	Included
Miller et al.^10^	Y	Y	Y	Y	Y	Y	Y	Y	Included
Simsek et al.^11^	Y	Y	Y	Y	Y	Y	N	Y	Included
Koo et al.^12^	Y	Y	Y	Y	Y	Y	N	Y	Included
Kyi et al.^13^	Y	Y	Y	Y	Y	Y	N	Y	Included
Naganuma et al.^14^	Y	Y	Y	Y	Y	Y	N	Y	Included
Dupuis et al.^15^	Y	Y	Y	Y	Y	Y	N	Y	Included
Rocha et al.^16^	Y	Y	N	Y	Y	Y	N	Y	Included
Legriel et al.^17^	Y	Y	Y	Y	Y	Y	N	Y	Included
Sakuragi et al.^18^	Y	Y	Y	Y	Y	Y	N	Y	Included

Tables 16 and 17 show the quality assessment of included case reports and case series as per Joanna Briggs Institute Critical Appraisal.^25^

**Table-XVI:** JBI critical Appraisal checklist for the included case series, where Y: Yes and N: No.

Author	Q1	Q2	Q3	Q4	Q5	Q6	Q7	Q8	Q9	Q10	Overall Appraisal
Lemke et al.^19^	N	Y	Y	N	Y	Y	Y	Y	Y	N	Included

### Clinical presentation of TCM:

The presentation of TCM is similar to acute coronary syndrome (ACS). According to literature chest pain and dyspnea are the most common presentation symptoms in patients of TCM.^7^ However, it has been observed that chest pain is an uncommon complaint in post-epileptic TCM.^16^ This aligns with findings in our study where a majority of patients presented with generalized tonic-clonic seizures or in the postictal state. Rocha et al. suggested that any subtle sign of cardiac distress could be an early indicator of the development of TCM in post-epileptic seizures.^16^ During hospital stay the most prominent signs depicting cardiac involvement were hypotension and tachycardia (Table-III).

### Diagnostic Criteria:

Echocardiography findings which help to support the diagnosis of Takotsubo cardiomyopathy include classical apical ballooning with basal hyperkinesia, reduced ejection fraction, mild mitral regurgitation and left ventricular outflow tract obstruction.^3^ This is similar to findings in this review where most patients had reduced ejection fraction of (15- 51%), hypokinesis/ apical ballooning and hyperkinetic basal segment (Table-IX). The most commonly used criteria for TCOM are modified Mayo Clinic diagnostic criteria which includes the following four components.

1. Temporary hypokinesis, akinesis or dyskinesis of the LV mid segments with or without apical contribution. The regional wall motion abnormalities that classically extend beyond a single coronary artery supply;
2. Lack of obstructive coronary artery disease (CAD) or angiographic proof of acute plaque rupture;
3. New ECG changes (either ST-segment elevations and/or T wave inversions) or modest rise in cardiac enzyme troponin-I;
4. No identifiable pheochromocytoma or myocarditis.^24^

### Evaluation of cardiac enzymes and ECG findings:

ECG can quickly assess any myocardial injury specifically during hemodynamic compromise.^8^ According to a study most common ECG abnormality in TCM was ST-elevation (82%) and showed T-wave inversion (64%) similar to findings in this study where predominant ECG findings were ST-segment elevation (38.88%), tachycardia (38.88%) and T wave inversion (28.88%) (Table-VI). Diffuse T wave inversions were seen in about 40% of the patients with TCM secondary to epileptic seizures in another study. This finding is often overlooked by clinicians or attributed to the effects of mediation causing a delay in the diagnosis of TCM.^9^

In this study, 83.33% of patients showed raised troponin levels with an average level of 7.14 ng/ml (Table 10). However, the levels are less than those seen in a typical ST elevation MI.^3^ These troponin levels are also observed to be disproportionate to cardiac dysfunction (less elevated comparatively).^19^ Similarly literature review suggests that BNP and N-terminal pro-BNP levels were elevated beyond what we typically encounter in MI.^3^ These findings correlate with study by Laghari et al. suggesting cardiac markers of myonecrosis (myoglobin, creatine kinase and troponin I and T) are raised to lesser degrees comparatively in TCM patients.^24^ This correlation signifies the monitoring of pro-BNP levels and trop I in patients developing cardiac symptoms after an episode of seizure.

### Types of Seizures associated with TCM:

In the literature, the most common types of seizure associated with TCM were generalized tonic-clonic seizure and complex partial seizure.^6^ This aligns with the finding of this study highlighting generalized tonic-clonic seizures as the most common seizures associated with TCM (Table-XI). However, it should also be kept in mind that patients present with prolonged episodes of generalized tonic-clonic seizure (status epilepticus) are at higher risk of TCM compared to patients exposed to brief episodes of seizure due to prolonged exposure to physical stress and circulatory catecholamines.^17^

### Previous medical history/ Risk factor:

Saouma et al. suggested that 75% of cases with TCM had an underlying neurological disease or epilepsy as a cause of TCM.^6^ Other neurological disease include subarachnoid hemorrhage (SAH), ischemic stroke and migraine.^7^ Similarity this study shows pre-existing epilepsy in 38.88% of patients and development of seizure secondary to ICH in 22.22% of patients. Hypertension and psychiatric disease remain the other significant risk factors in past medical history (Table-V). It is important to note that new-onset epilepsy secondary to a vascular lesion, post-traumatic lesion or tumor has been documented as the reason for the development of TCM.^13^

The study by Kyi et al. reported 27% of patients developed TCM secondary to emotional distress, 38% by physical trigger and 28.5% had no triggers at all 13 making non-neurological factors the majority of causes in the development of TCM. However, it has been observed that nearly all case reports lack the element of psychological assessment which is an established contributing factor in the development of TCM.

### Progressive course of TCM:

The mortality rate of TCM is up to 8%. In literature, full recovery has been reported in 97% of cases and fatal outcomes observed in 3% of cases.^13^ This is similar to our study complete resolution of echocardiographic changes (55.55%) and full recovery has been observed. However, 16.66% developed cardiogenic shock and pulmonary oedema (Table-XII). Progression of TCM puts the patient at risk of ventricular wall rupture, cardiogenic shock, heart failure and arrhythmia.^8^

### Treatment:

In our study patients have been managed for neurological as well as cardiac symptoms. For neurological symptoms i.e. seizures, Benzodiazepines are used in 50% of patients, most commonly used benzodiazepines were Diazepam (22.22%). Other drugs were Phenytoin (38.88%), Sodium valproate (33.33%) and Levetiracetam (27.77%) (Table-XIII). For the management of cardiac symptoms, B-blockers are used in 61.11% of patients, angiotensin-converting enzyme inhibitors (ACE-I) in 33.33% of patients and inotropes in 22.22% of patients. This is similar to the study by Amin et al. according to which treatment of TCM with heart failure drugs include angiotensin-converting enzyme inhibitors (ACE-I) or angiotensin receptor blockers (ARB) and beta blockers which show a good clinical response.^1^ Furthermore, initiation of anticoagulation therapy might be useful to prevent thrombosis and embolic events once TCM diagnosis is confirmed. However, the mainstay of treatment relies on stabilizing the patient with cardiopulmonary supportive care on presentation along with anti-epileptic medications to stop the ongoing seizure activity.^1^ However, in a study by Laghari et al. 58.62% of patients experiencing cardiogenic shock due to severe left ventricular dysfunction, intra-aortic balloon pump (IABP) was placed. IABP is a preferred treatment in patients with severe left ventricular dysfunction.^24^ Overall, after treatment 55.55% showed improvement in their echocardiographic findings and 11.11% showed ECG findings (Table-XV)

### Limitations:

When discussing the limitations of our review, it’s essential to acknowledge the constraints posed by the dataset utilized. Our review’s dataset comprised a total of 18 patients, derived from 15 case reports and a single case series. This relatively small sample size, coupled with the reliance on case reports and series, limits the generalizability of our findings. Case reports and series, while valuable for highlighting unique or novel presentations, may not provide the comprehensive data or the controlled environments that larger randomized controlled trials offer. Therefore, while our review offers insights into the relationship between Takotsubo cardiomyopathy and epilepsy, these findings should be interpreted with caution, considering the potential for selection bias and the lack of broader epidemiological data. Future studies with larger, more diverse populations and controlled study designs are needed to validate and expand upon our findings.

## CONCLUSION

In conclusion, our investigation into the pathophysiological mechanism of Takotsubo cardiomyopathy (TCM) in epilepsy patients has illuminated the critical role of excessive catecholamine release following seizure. This surge in catecholamines, particularly complex partial and generalized tonic-clonic seizures, appears to precipitate TCM by including transient myocardial dysfunction. This study underscores the predominance of TCM in postmenopausal women, suggesting a potential hormonal influence on the heart’s sensitivity to catecholamines.

The findings also emphasize the importance of monitoring troponin and BNP levels in the acute post-ictal period to promptly identify and manage TCM. This approach not only aims to mitigate the immediate cardiac risk associated with TCM but also addresses the long-term goal of reducing frequency and severity through optimized antiepileptic therapy.

Future research should focus on unraveling the detailed molecular mechanism linking seizure to cardiac dysfunction and exploring gender-specific responses in the pathogenesis of TCM. By enhancing our understanding of this mechanism, we can improve diagnostic accuracy, tailor therapeutic interventions more effectively, and ultimately improve the prognosis for epilepsy patients at risk of TCM.

### Clinical Recommendations:

Based on our findings, we recommend that physicians maintain a high index of suspicion for TCM in patients with epilepsy, especially following a seizure. Given the potential for cardiac complications in these patients, it’s advisable to monitor cardiac function closely in the acute postictal period. Electrocardiographic monitoring and echocardiography may be particularly useful in detecting transient cardiac abnormalities characteristic of TCM. In managing patients with epilepsy who are at risk for TCM, stress reduction strategies and careful seizure management are paramount. Collaborative care between neurologists and cardiologists to tailor treatment plans that address both seizure control and cardiovascular health can help to reduce risk of recurrence. Awareness of the potential for TCM in epilepsy patients can enhance early detection and intervention, potentially improving patient outcomes.

### Authors Contribution

**HMQ** conceptualized and designed the study, did literature review, analyzed data and critical review of the manuscript.

**FI** literature review and study, compilation and acquisition of data and manuscript drafting.

**MK, AS and SB** literature study, acquisition of data and manuscript drafting.

**AB** supervised the study, critical review of the manuscript and data analysis.

All authors are responsible and accountable for the accuracy or integrity of the work.
